# Comparison of the therapeutic effects of three minimally invasive approaches for laparoscopic cholecystectomy combined with common bile duct exploration—— a 5-year retrospective analysis

**DOI:** 10.1186/s12893-024-02490-4

**Published:** 2024-07-02

**Authors:** Liqiang Li, Zihan Zeng, Liang Li, Jun Zhang

**Affiliations:** 1 Department of General Surgery, the Second People’s Hospital of Hefei or Hefei Hospital Affiliated to Anhui medical University, Hefei, 230011 China; 2 Second People ’ s Hospital, Bengbu Medical College, Hefei, 230011 China; 3 Hefei Hospital Affiliated to Bengbu Medical University, Hefei, 230011 China

**Keywords:** Common bile duct stones, Primary closure, Double J-tube, Internal/external drainage

## Abstract

**Objective:**

The aim of this retrospective study was to explore the indications for three minimally invasive approaches—T-tube external drainage, double J-tube internal drainage, and primary closure—in laparoscopic cholecystectomy combined with common bile duct exploration.

**Methods:**

Three hundred eighty-nine patients with common bile duct stones who were treated at the Second People's Hospital of Hefei between February 2018 and January 2023 were retrospectively included. Patients were divided into three groups based on the surgical approach used: the T-tube drainage group, the double J-tube internal drainage group, and the primary closure group. General data, including sex, age, and BMI, were compared among the three groups preoperatively. Surgical time, length of hospital stay, pain scores, and other aspects were compared among the three groups. Differences in liver function, inflammatory factors, and postoperative complications were also compared among the three groups.

**Results:**

There were no significant differences among the three groups in terms of sex, age, BMI, or other general data preoperatively (*P* > 0.05). There were significant differences between the primary closure group and the T-tube drainage group in terms of surgical time and pain scores (*P* < 0.05). The primary closure group and double J-tube drainage group differed from the T-tube drainage group in terms of length of hospital stay, hospitalization expenses, and time to passage of gas (*P* <0.05). Among the three groups, there were no statistically significant differences in inflammatory factors or liver function, TBIL, AST, ALP, ALT, GGT, CRP, or IL-6, before surgery or on the third day after surgery (*P* > 0.05). However, on the third day after surgery, liver function in all three groups was significantly lower than that before surgery (*P*<0.05). In all three groups, the levels of CRP and IL-6 were significantly lower than their preoperative levels. The primary closure group had significantly lower CRP and IL-6 levels than did the T-tube drainage group (*P* < 0.05). The primary closure group differed from the T-tube drainage group in terms of the incidences of bile leakage and electrolyte imbalance (*P* < 0.05). The double J-tube drainage group differed from the T-tube drainage group in terms of the tube dislodgement rate (*P *< 0.05).

**Conclusion:**

Although primary closure of the bile ducts has clear advantages in terms of length of hospital stay and hospitalization expenses, it is associated with a higher incidence of postoperative complications, particularly bile leakage. T-tube drainage and double J-tube internal drainage also have their own advantages. The specific surgical approach should be selected based on the preoperative assessment, indications, and other factors to reduce the occurrence of postoperative complications.

Common bile duct stones are a common disease of the extrahepatic biliary system and are divided into primary and secondary types [1]. Typically, patients with bile duct stones do not experience abdominal pain, nausea, vomiting, or other upper gastrointestinal discomfort when the stones are not causing obstruction [2]. However, when bile duct stones cause obstruction, secondary bile duct infection quickly follows, leading to symptoms of acute cholangitis, such as abdominal pain, high fever, and jaundice, which can be fatal [3]. With the aging of the population, the incidence of this disease is increasing, with a prevalence rate of up to 30% in elderly patients [4]. Traditionally, open surgery is performed to remove common bile duct stones [5]. However, laparoscopic common bile duct exploration and stone extraction have become common surgical approaches. It is still unclear whether primary closure of the common bile duct should be performed, or whether a T-tube or a double J-tube should be inserted after common bile duct exploration and stone extraction [6–8]. The aim of this study is to explore the different options for managing the common bile duct during laparoscopic exploration and stone extraction, as well as postoperative complications, in patients who undergo this procedure.

## Data and methods

### Clinical data

A total of 389 patients with common bile duct stones who were treated between February 2018 and January 2023 were retrospectively enrolled. The patients were divided into three groups based on the surgical approach used: the T-tube drainage group, the double J-tube internal drainage group, and the primary closure group. There were no statistically significant differences among the three groups in terms of age, sex, number of stones, diameter of the bile duct, BMI, transaminase levels, bilirubin levels, or other parameters (*P* > 0.05) (Table 1).

**Table 1:** Preoperative general data of the patients in the three groups (¯X ± s)

Data	T-tube drainage group (*n* = 139)	Double J-tube internal drainage group (*n* = 122)	Primary closure group (*n* = 128)	χ^2^/t	*P*
Gender [n(%)]					
Male	72(51.8%)	69(56.56%)	76(59.34%)	0.247	0.382
Female	67(48.20%)	53(43.44%)	52(40.62%)		
Age (years)	54.27 ± 12.92	57 ± 14.29	52 ± 12.27	0.429	0.272
BMI(kg/m^2^)	22.82 ± 2.23	22.37 ± 2.24	20.29 ± 2.39	0.349	0.242
Number of stones	2.3 ± 0.40	2.4 ± 0.20	2.3 ± 0.20	0.149	0.086
Stone diameter	1.10 ± 0.13	1.14 ± 0.12	1.12 ± 0.22	1.493	0.276
Bile duct diameter	1.1 ± 0.10	1.3 ± 0.30	1.3 ± 0.10	1.727	0.159
Whether jaundice is present (Yes/No)	59/80	48/74	46/82	0.324	0.279
Preoperative CA19-9 level	42.59 ± 10.27	40.11 ± 12.09	41.29 ± 10.69	0.159	0.837
ALT (U/L)	187.59 ± 75.28	218.8 ± 82.49	189.29 ± 62.39	1.276	0.393
TBIL(µmol/L	64.81 ± 2.42	69.74 ± 1.90	52.86 ± 1.51	1.638	0.289

### Inclusion criteria

① Preoperative imaging examinations, such as abdominal ultrasound and MRCP, confirmed the presence of common bile duct stones with a diameter exceeding 8 mm. ② Experienced biliary colic at least once in the past year. ③ Patency of the distal common bile duct and normal function of the Oddi sphincter with no distal strictures.

### Exclusion criteria

① Clinical, laboratory, and imaging findings suggestive of acute pancreatitis or cholangitis. ② Imaging evidence of liver abscess, cirrhosis, or other tumor diseases. ③A history of abdominal surgery or severe underlying conditions not suitable for any invasive procedures.

All patients were informed of the study details and signed informed consent forms. The participants and/or their legal guardians were adequately informed. This study was approved by the Ethics Committee of our hospital. Postoperative follow-up, including telephone follow-up and outpatient visits, was conducted at 18–22 months.

### Methods

**Fig. 1:**
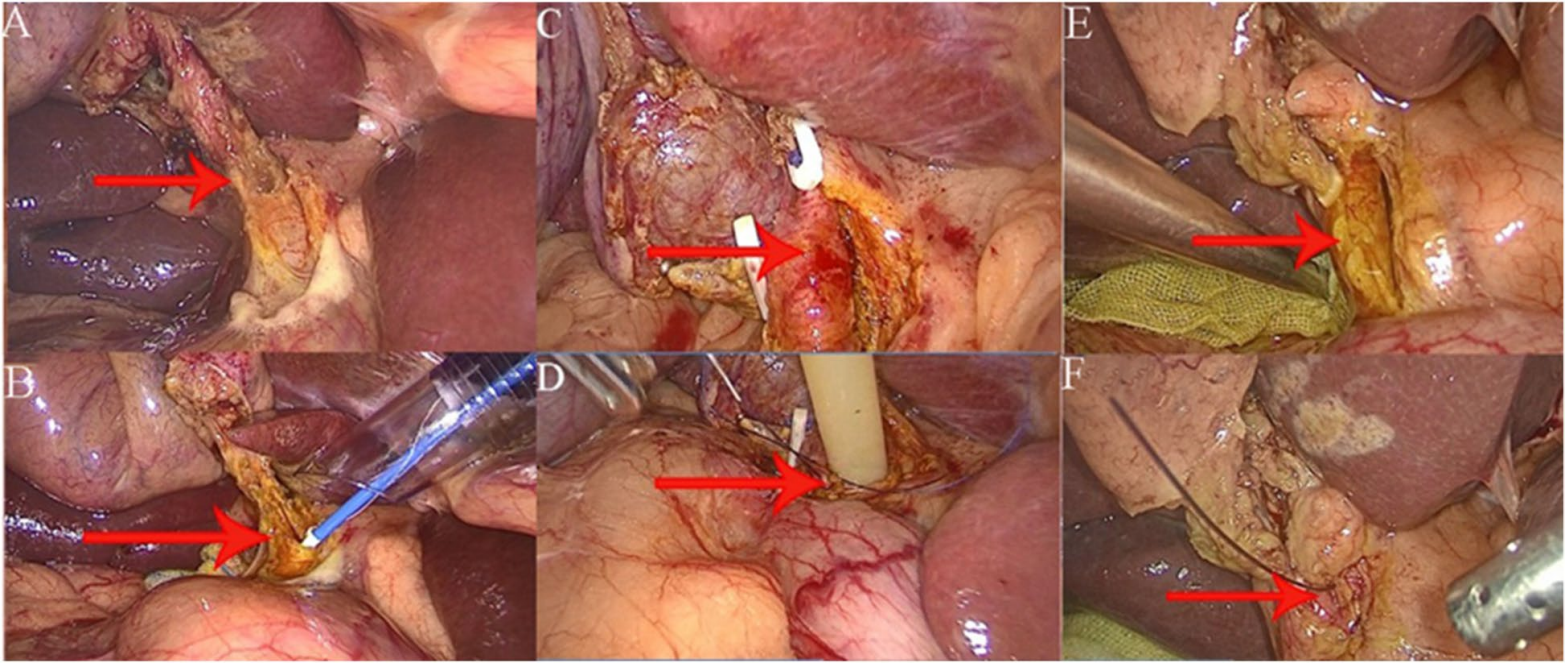
**A**-**B **Double J stent drainage; **C**-**D **Common bile duct T-tube drainage; **E**-**F **Primary common bile duct repair

Tracheal intubation was performed, intravenous anesthetics were administered, and the intra-abdominal pressure was maintained at 10–12 mmHg. A conventional four-port method was used for laparoscopic access. The gallbladder triangle was dissected, and the cystic duct was freed, leaving it temporarily uncut approximately 0.5 cm from the common bile duct to prevent small stones from being squeezed into the common bile duct and to facilitate intraoperative traction. After exposing the common bile duct, a longitudinal incision measuring approximately 1.0 cm in length was made on the anterior wall of the common bile duct, and an electronic choledochoscope was inserted for exploration. Stones were retrieved using a stone retrieval basket. In cases of difficult stone retrieval, lithotripsy was performed with a liquid-electric lithotripter before stone retrieval. Choledochoscopy was repeatedly performed to confirm the absence of residual stones in the intra- and extrahepatic bile ducts and the absence of distal bile duct stricture. Patients were divided into three groups according to the method of management used. In the J-tube internal drainage group (Fig. 1A), a 7# double J-tube that is commonly used in urology was guided into the duodenum under choledochoscopy. After ensuring that the distal circle of the double J-tube entered the duodenum and confirmed its entry into the intestine with the choledochoscope, more than half of the proximal double J-tube was cut off and inserted into the right hepatic duct. Interrupted sutures with 4 − 0 Vicryl suture threads were used to close the incision in the common bile duct. In the T-tube external drainage group (Fig. 1C), a 20# T-tube was slowly inserted into the common bile duct, and the distal end was clipped to prevent bile leakage. Interrupted sutures with 4 − 0 Vicryl suture threads were used to close the incision in the common bile duct. In the primary closure group (Fig. 1E), no bile duct drainage devices were used. The incision in the common bile duct was directly closed with interrupted sutures using 4–0 Vicryl suture threads. After completely closing the bile duct, cholecystectomy was performed, and a drainage tube was routinely placed at the Winslow foramen. All surgeries were performed by the same deputy chief physician in the hepatobiliary surgery department of our hospital, and regular outpatient follow-ups were conducted.1.5 Outcome Measures: The analysis included common demographic data such as gender, age, and stone diameter. Intraoperative parameters such as surgical time and intraoperative blood loss were compared, as well as postoperative outcomes including time to resume eating and length of hospital stay. Additionally, postoperative complications and long-term recurrence rates between the two groups were compared. All surgeries were conducted according to relevant guidelines and protocols.

## Statistical analysis

Statistical analysis was performed using SPSS 22.0 software. Continuous data are expressed as the mean ± standard deviation (¯X ± s), and comparisons were made using the t test. Categorical data are presented as counts (percentages), and comparisons were conducted using the chi-square test or Fisher's exact test. A *P* value < 0.05 was considered to indicate statistical significance.

## Results

The preoperative general data, including sex, age, BMI, stone diameter, and presence of jaundice, were not significantly different among the three groups of patients (*P* > 0.05) (Table 1).

There were significant differences between the primary closure group and the T-tube drainage group in terms of operation time and pain scores (*P* < 0.05); significant differences were also noted between the primary closure group and the double J-tube drainage group in terms of length of hospital stay, hospital costs, and time to flatus compared to the T-tube drainage group (*P*< 0.05) (Table 2).

**Table 2:** Comparison of the surgical characteristics among the three groups of patients (¯X ± s)

Data	T-tube drainage group (*n* = 139)	Double J-tube internal drainage group (*n* = 122)	Primary closure group (*n* = 128)	χ^2^/t	*P*
Surgical time (minutes)	118.51 ± 5.92	92.42 ± 6.20^a^	90.59 ± 4.7^a^	1.729	0.018
Length of hospital stay (days)	8.50 ± 0.70	5.20 ± 0.20^a^	4.70 ± 0.40^a^	32.58	<0.01
Time to gas passage (days)	4.76 ± 0.45	3.59 ± 1.29^a^	2.27 ± 1.12^a^	1.59	0.027
Hospital costs (CNY)	25017 ± 425.82	21127 ± 495.34^a^	17529 ± 526.8^a^	45.272	<0.01
Pain score	2.2 ± 1.2	1.9 ± 0.7	1.5 ± 0.6^a^	1.29	<0.01

Compared with the T-tube drainage group^a^*P*<0.05

Before surgery and on the third postoperative day, there were no statistically significant differences among the three groups of patients in terms of TBIL, AST, ALP, ALT, GGT, CRP, or IL-6 levels (*P* > 0.05). On the third postoperative day, the liver function parameters TBIL, AST, ALP, ALT, and GGT were lower than the preoperative levels in all three groups of patients. Additionally, in the primary closure group, the TBIL, AST, ALP, ALT, and GGT levels were significantly lower than those in the T-tube drainage group (*P *< 0.05). Furthermore, the postoperative inflammatory markers CRP and IL-6 were lower than the preoperative levels in all three groups of patients. In the primary closure group, both the CRP and IL-6 levels were significantly lower than those in the T-tube drainage group (*P* < 0.05) (Table 3).

**Table 3:** Changes in liver function and inflammatory markers before and after surgery among the three groups of patients (¯X ± s)

Groups	Time	TBIL(µmol/L)	AST(U/L)	ALP(U/L)	ALT(U/L)	GGT(U/L)	CRP(mg/L)	IL-6(pg/ml)
	Preoperative	64.81 ± 2.42	195 ± 59.23	349.29 ± 49.56	187.59 ± 75.28	109.69 ± 7.19	72.19 ± 7.21	92.34 ± 7.12
T-tube drainage group (*n* = 139)								
	Postoperative day 3	20.75 ± 6.79^a^	58.39 ± 37.28^a^	85.39 ± 23.63^a^	47.29 ± 53.26^a^	86.39 ± 6.46^a^	50.28 ± 5.19^a^	25.28 ± 6.27^a^
t		5.372	2.283	14.28	2.292	10.22	11.23	5.32
*P*		0.000	0.013	0.000	0.022	0.000	0.000	0.000
	Preoperative	69.74 ± 1.90	193.28 ± 49.83	372.26 ± 39.28	218.8 ± 82.49	113.22 ± 7.21	73.27 ± 6.19	89.38 ± 6.73
Double J-tube internal drainage group (*n* = 122)								
	Postoperative day 3	48.29 ± 1.4^a^	53.87 ± 32.73^a^	82.37 ± 21.49^a^	42.46 ± 49.22^a^	81.29 ± 5.92^a^	52.39 ± 5.33^a^	36.27 ± 6.39^a^
t		4.429	2.351	12.29	2.29	14.39	13.49	4.38
*P*		0.000	0.000	0.000	0.000	0.000	0.000	0.000
	Preoperative	62.86 ± 1.51	227.29 ± 39.38	389.39 ± 27.59	189.29 ± 62.39	119.39 ± 6.39	79.38 ± 7.28	79.86 ± 5.39
Primary closure group (*n* = 128)								
	Postoperative day 3	42.39 ± 1.49^ab^	47.29 ± 28.38^ab^	79.29 ± 32.59^ab^	47.29 ± 59.38^a^	79.28 ± 5.35^ab^	49.49 ± 5.38^a^	33.29 ± 4.59^ab^
t		5.24	4.462	2.496	12.28	2.46	13.29	12.39
*P*		0.000	0.000	<0.01	0.000	<0.01	0.000	<0.01
T^Three groups on the third day after surgery^		3.26	5.223	3.462	2.75	3.53	7.04	6.42
*P* ^Three groups on the third day after surgery^		<0.05	<0.01	<0.05	0.128	<0.05	0.283	<0.05

Compared with the preoperative values within the same group, ^a^*P* < 0.05; compared with the T-tube drainage group on postoperative day 3, ^b^*P* < 0.05

The primary closure group significantly differed from the T-tube drainage group in terms of the incidences of bile leakage and electrolyte disturbances (*P* < 0.05). Additionally, the incidence of tube dislodgement significantly differed between the double J-tube internal drainage group and the T-tube drainage group (*P* < 0.05) (Table 4).

**Table 4:** Comparison of postoperative complications among the three groups (¯X ± s)

Data	T-tube drainage group (*n* = 139)	Double J-tube internal drainage group (*n* = 122)	Primary closure group (*n* = 128)	*F*	*P*
Bile leakage	1	1	5^a^		*P*<0.05
Residual stones	1	2	2		0.549
Biliary stricture	0	0	0	—	—
Biliary bleeding	1	1	0		—
T/Double J-tube dislodgement	7	2^b^	0		0.018
Electrolyte disturbances	12	3	1^a^		0.001

a/b compared with the T-tube drainage group, *P* < 0.05

## Discussion

The goal of treatment for extrahepatic bile duct stones is complete removal of bile duct stones and pain relief. Currently, there are various methods available for stone removal, with ERCP combined with EST being a representative approach [9, 10]. This method is advantageous in that most bile duct stones can be cleared by incising the sphincter of the ampulla of Vater without the need for T-tube placement [11, 12]. However, the disadvantages of this approach may include postoperative complications such as pancreatitis, bleeding, and perforation. Furthermore, in some cases, long-term bile reflux caused by an incision in the sphincter of the ampulla of Vater may lead to the recurrence of bile duct stones and the development of biliary tract tumors [13, 14]. Laparoscopic common bile duct exploration (LCBDE), which involves laparoscopic choledochotomy for stone removal combined with choledochoscopy, has emerged as an advantageous approach [15]. Studies have indicated that LCBDE is often a preferred option in many cases [14]. However, conventional LCBDE typically involves routine placement of a T-tube, which can lead to complications such as bile leakage, electrolyte disturbances, or T-tube dislodgement postoperatively [8, 16–18]. Moreover, these T-tubes often need to be kept in place for more than 4 weeks, during which patients are prone to experiencing electrolyte disturbances and T-tube dislodgement complications [19, 20]. Additionally, patients require a second hospitalization for T-tube removal, which adds psychological, physiological, and financial burdens. Consequently, some scholars have proposed primary closure of the bile duct [21].

The results of this study suggest that primary closure of the bile duct appears to be superior to T-tube drainage in terms of surgical time, length of hospital stay, and hospital costs, indicating that it is potentially the optimal surgical approach. However, analysis of postoperative complications revealed that patients in the primary closure group had the highest incidence of bile leakage. Further investigation revealed that this occurrence was primarily due to an excessive number of stones within the bile duct [15]. The occurrence of such complications is attributed to repeated manipulation of the sphincter of the ampulla of Vater during stone extraction, resulting in spasms, edema, and even bleeding [22]. The blood clot caused by bleeding blocks the lower end of the bile duct, leading to the obstruction of bile excretion. Moreover, the compensatory dilation capacity of the smaller bile ducts is limited, ultimately resulting in increased intrabile duct pressure and bile leakage. Therefore, primary closure is suitable for patients with a bile duct diameter > 10 mm and no inflammation, edema, or stenosis at the lower end of the bile duct [23].

With the development of minimally invasive surgery and advancements in surgical techniques, urological double J tubes can be applied to the common bile duct to serve as supportive stents. The use of double J tubes or other plastic stents for internal biliary drainage combined with primary closure can effectively avoid the drawbacks of T-tube drainage. The results of this study indicate that patients with retained T-tubes had the highest incidence of electrolyte disturbances among the three surgical methods. Compared to T-tube drainage, double J-tube drainage is more physiologically functional, reducing electrolyte disturbances caused by bile loss and improving patients' quality of life. However, the placement of urological double J tubes also increases a patient's financial burden, which may cause economic discomfort. Additionally, in some cases, the inserted double J tube cannot be spontaneously expelled and requires removal via gastroscopy, which is a disadvantage of double J tube use [24].

Although T-tube drainage showed average results in terms of length of hospital stay, hospital costs, and T-tube dislodgement rates in this study, it demonstrated excellent results in terms of the stone clearance rate and prevention of bile leakage. We believe that the larger diameter of the T-tube compared to the diameter of the double J-tube allows for better bile drainage and a reduction in intrabile duct pressure, thereby resulting in a lower incidence of postoperative bile leakage. This external drainage method is suitable for patients with a large number of intrabile duct stones who require multiple stone removal procedures, as well as those with severe symptoms of bile duct inflammation and significant bile duct wall edema [15]. Studies have indicated that T-tube drainage is particularly suitable for patients who have undergone secondary biliary tract surgery or who have a history of abdominal surgery [23].

It is important to emphasize that this study does not advocate for any particular surgical approach, nor does it negate other surgical methods. Regardless of the surgical approach, patients showed significant improvement in liver function and inflammatory markers on the third postoperative day, which is consistent with the results of this study. Therefore, the choice of surgical approach should be based on the characteristics of the patient and the experience of the surgeon.

In summary, although primary closure of the bile duct has advantages in terms of length of hospital stay and hospital costs, it is associated with higher incidences of postoperative bile leakage and residual stones. Both T-tube drainage and double J-tube drainage have their respective advantages and indications. Therefore, the choice of surgical approach should be based on factors such as the patient's financial status, indications, and preoperative imaging assessment.

## Data Availability

The datasets generated during and/or analyzed during the current study are not publicly available, due to the involvement of patients’ personal privacy, the data cannot be uploaded to the database at the moment but are available from the corresponding author on reasonable request.
